# Comparative analysis of regression algorithms for drug response prediction using GDSC dataset

**DOI:** 10.1186/s13104-024-07026-w

**Published:** 2025-01-13

**Authors:** Soojung Ha, Juho Park, Kyuri Jo

**Affiliations:** Department of Computer Engineering, Chungbuk National University, Chungdae-ro 1, Cheongju, 28644 Republic of Korea

**Keywords:** Drug response, Regression, Gene expression, Multiomics, GDSC dataset

## Abstract

**Background:**

Drug response prediction can infer the relationship between an individual’s genetic profile and a drug, which can be used to determine the choice of treatment for an individual patient. Prediction of drug response is recently being performed using machine learning technology. However, high-throughput sequencing data produces thousands of features per patient. In addition, it is difficult for researchers to know which algorithm is appropriate for prediction as various regression and feature selection algorithms exist.

**Methods:**

We compared and evaluated the performance of 13 representative regression algorithms using Genomics of Drug Sensitivity in Cancer (GDSC) dataset. Three analyses was conducted to show the effect of feature selection methods, multiomics information, and drug categories on drug response prediction.

**Results:**

In the experiments, Support Vector Regression algorithm and gene features selected with LINC L1000 dataset showed the best performance in terms of accuracy and execution time. However, integration of mutation and copy number variation information did not contribute to the prediction. Among the drug groups, responses of drugs related with hormone-related pathway were predicted with relatively high accuracy.

**Conclusion:**

This study can help bioinformatics researchers design data processing steps and select algorithms for drug response prediction, and develop a new drug response prediction model based on the GDSC or other high-throughput sequencing datasets.

**Supplementary Information:**

The online version contains supplementary material available at 10.1186/s13104-024-07026-w.

## Introduction

As the era of precision medicine commences, a number of studies are seeking to utilise the genomic profiles of cancer patients in order to design personalised treatments [1]. Notwithstanding the advent of an array of cancer therapies, drug treatments remain susceptible to influence from genetic and environmental conditions. In other words, the effects of a given drug can vary depending on the interaction between the drug and the molecules present in the body [2]. It is therefore imperative to predict the effectiveness of drugs using genomic information and to enhance therapeutic responses.

One of the research topics in pharmacogenomics is drug response prediction. This aims to predict the sensitivity of each genome to drugs using diverse genomic information, including single nucleotide variations, copy number variations, RNA expression, methylation, and proteomics [3]. In order to achieve accurate predictions of drug response, it is essential to generate and utilise large-scale datasets in predictive models. The Genomics of Drug Sensitivity in Cancer (GDSC) represents one of the most comprehensive pharmacogenetic datasets, initially released in 2012. It encompasses assay data for drug sensitivity across 969 human cancer cell lines and 297 compounds, including 243,466 IC50 values [4].

The prediction of drug responses can be modelled as a regression problem, and a variety of regression algorithms can be applied to drug sensitivity data. However, many of these techniques rely on sophisticated algorithms that may not be readily accessible to researchers lacking a strong computational background. In contrast, the basic regression models currently implemented in Python libraries such as Scikit-Learn [5] are readily accessible for researchers in the biological and bioinformatics fields. This study deliberately focuses on these accessible tools with the objective of providing a practical framework that can be readily implemented by researchers from a range of fields, including those who may lack advanced computational expertise. While a number of regression algorithms are available, researchers may find it challenging to identify the most appropriate algorithm for drug response prediction and to preprocess the data, given the extensive number of input features typically present in genomics data.

In this study, 13 representative regression algorithms were employed for the purpose of predicting drug sensitivity using the GDSC dataset. The objective of this research is to provide clear and accessible guidelines for bioinformatics researchers in the design of data processing steps and the selection of algorithms for drug response predictions, while also considering predictive accuracy.To this end, three analyses were devised. Firstly, a comparison was conducted between four feature selection methods, comprising three existing algorithms and one based on biological experiments. Secondly, the impact of integrating multi-omics data, including somatic mutation and copy number variation, into gene expression datasets was demonstrated. Thirdly, the accuracy of the predictions was compared across different drug groups.

## Methods

### Data sets

In order to train machine learning models, genomic profiles and IC50 values for drug response markers were retrieved from [6]. The dataset comprises 8,046 genes from a total of 734 cancer cell lines. The genomic profiles employed in this study comprise gene expression, copy number variation (CNV), and mutation datasets derived from the GDSC dataset. The aforementioned datasets were directly obtained from the study by Chen and Zhang, where the preprocessing of the genomic profile data was described in Section 4.1 of [6].

The gene expression data are arranged in a matrix comprising 734 rows and 8046 columns, representing 734 cancer cell lines and 8046 genes, respectively. The gene expression data for each cell line is meticulously catalogued and identified using the COSMIC ID. In addition to gene expression data, other omics data, including mutation and copy number variation (CNV) profiles, were also incorporated as input features to investigate the impact of integrating multi-omics features. The mutation data are structured in a matrix of 734 x 636, in which the rows represent the 734 cell lines (identified by COSMIC ID) and the columns represent the genes. The presence or absence of a mutation is indicated by a binary value, with 0 denoting the absence of a mutation and 1 indicating its presence. Similarly, the CNV data are organised in a matrix of 734 x 694, encompassing the CNV status for genes in 734 cell lines (identified by COSMIC ID). The CNV status is represented by binary values, with a value of 0 indicating a normal copy number and a value of 1 indicating a variation. A total of 636 and 694 features were derived from mutation and copy number variation profiles, respectively, as in [6].

Furthermore, information on drug groups was incorporated into this study. As described in [6], the 201 drugs were classified into 23 groups based on their targeted pathways.

### Regression algorithms used

The following Python-based 13 regression algorithms, included in the scikit-learn library, were tested in an experimental setting. Each algorithm is classified into one of six categories, as outlined in Table 1. The regression algorithms employed in this study align with the fundamental regression algorithms utilized in machine learning. The objective is to provide training guidelines for basic machine learning regression models to biologists and bioinformatics researchers who wish to use the GDSC dataset. The intention is to implement regression models that are relatively accessible.

The Elastic Net, LASSO, Ridge, and SVR algorithms are based on the principles of linear regression. These algorithms employ linear relationships between input features and weights to predict continuous outputs. With the exception of SVR, the remaining three algorithms utilise L1 and L2 rules to reduce model complexity. Support vector regression (SVR) employs support vectors to establish a linear relationship between input features and outputs.

The algorithms designated ADA, DTR, GBR, RFR, XGBR and LGBM are decision tree-based and utilise input features for the purpose of data segmentation and predictive modelling. In this process, a series of decision trees are constructed, and weights are assigned to each tree. Alternatively, the trees are learned in a sequential manner to create a predictive model. One advantage of this approach is that it allows the selection of an appropriate model, according to the structure or complexity of the data.

MLP is an artificial neural network based on a multilayer perceptron. The network is composed of three layers: an input layer, a hidden layer, and an output layer. The neurons in each layer receive input through an activation function. The capacity to model intricate, non-linear relationships using multilayer structures represents a significant advantage of this approach, and it is therefore employed in a range of deep learning applications.

The K-nearest neighbour (KNN) algorithm is a type of machine learning that relies on the concept of nearest neighbours. The prediction is performed by tracking the K nearest neighbours of the given data, that is to say, the K data points that are most similar or closest to the given data. The value of K can be selected at the discretion of the user, and the neighbouring data are selected through the application of calculations such as the Euclidean distance and the Manhattan distance. The KNN algorithm can be used for both classification and regression, and offers an intuitive interface.

GPR offers forecasts for forthcoming data based on a Gaussian distribution. The method is effective for small datasets, but the accuracy of calculations is compromised for large datasets.

The parameters of all models employed in this study have been collated and are presented in a supplementary file (Additionl file 1 Table S1).

### Feature selection methods

The algorithms of mutual information (MI), variance threshold (VAR), and select K best features (SKB) from the Python scikit-learn library were selected as representative feature selection algorithms for gene expression data. As an additional feature selection method, the Library of Integrated Network-Based Cellular Signatures (LINCS) L1000 dataset was employed [7]. The L1000 library offers insight into the biological, genetic, chemical and medical reactivity of cells associated with various diseases, as well as a list of approximately 1,000 major genes that demonstrated a significant response during drug screening. The L1000 dataset indicates the use of 627 genes that are commonly present in both the GDSC and L1000 datasets as features of gene expression data.

**Table 1:** Summary of regression algorithms used in this study

Library/Algorithm	Abbreviation	Description	Category
sklearn/KNeighborsRegressor	KNN	Get an output which is the average property values of k nearest neighbors [8]	Miscellaneous
sklearn/RandomForestRegressor	RFR	Concept of regression trees by exploiting the power of computers to simultaneously generate hundreds of regression trees[9]	Ensemble
sklearn/SupportVectorRegressor	SVR	Characterized by the use of kernels, sparse solution, and VC control of the margin and the number of support vectors[10]	Kernel-based
sklearn/DecisionTreeRegressor	DTR	Generates a decision tree from given instances [11]	Tree- or rule-base
sklearn/AdaBoostRegressor	ADA	Consists of several decision tree regressors as a weak learner[12]	Ensemble
sklearn/GradientBoostingRegressor	GBR	Integrated model with higher performance and better stability [13]	Ensemble
lightgbm/LGBMRegressor	LGBM	Framework for implementing Gradient Boosting Decision Tree [14]	Ensemble
xgboost/XGBRegressor	XGBR	Scalable machine learning system for tree boosting [15]	Ensemble
sklearn/MLPRegressor	MLP	Feed-forward neural networks to deal with non-linear regression models [16]	Artificail neural network
sklearn/GaussianProcessRegressor	GPR	Nonparametric method that belongs to the Bayesian statistics family [17]	Miscellaneous
sklearn/Ridge	RGE	Designed to find the linear hyperplane that approximates the data labels well [18]	Regularized
sklearn/Lasso	LAS	Based on the concept of minimizing the standard mean squared error penalized by the sum of absolute values of the regression coefficients[19]	Regularized
sklearn/ElasticNet	EN	Form of regularized optimization for linear regression [20]	Regularized

### Evaluation metrics

The evaluation of regression model performance is typically conducted using a range of metrics, including Mean Squared Error (MSE), Mean Absolute Error (MAE), Root Mean Squared Error (RMSE), Coefficient of Determination (${\hbox {R}}^{2}$) and Correlation Coefficient.

In this study, MAE scores were employed. While MSE and RMSE are widely used evaluation metrics, they have inherent limitations in expressing the magnitude of changes, particularly in cases where the variation in magnitude is of major importance. We found MAE to be particularly well-suited to our objective of identifying an evaluation metric that provides a comprehensive representation of the distinctions between algorithms. MAE is a simple and straightforward expression of the average absolute deviation between the expected and actual values. MAE is an effective method for identifying the extent of divergence between algorithmic outcomes, irrespective of any scaling or square-root transformations that may be applied.

In order to guarantee the robustness of the evaluation, a three-fold cross-validation approach was employed. This method permits the evaluation of the models on disparate subsets of the data, thereby facilitating a more reliable estimation of their predictive performance. The dataset was divided into three equal portions, with two allocated for training and the remaining portion designated for validation. This process was repeated on three occasions, and the mean performance metrics were calculated in order to assess the models.

To facilitate a comparative analysis of feature selection algorithms, an analysis of variance (ANOVA) is conducted, with the R-squared value of each dataset serving as the primary metric. The objective of this study was to ascertain whether there were significant differences in the prediction results obtained from different feature selection algorithms using the ANOVA technique. This specific statistical method is particularly well-suited to the task at hand for a number of reasons. Firstly, this study involves comparing various feature selection methods and regression models. Evaluating the performance of each feature selection method applied to different regression models necessitates multiple group comparisons. ANOVA is specifically designed to handle such comparisons by analyzing the variance among group means and determining whether statistically significant differences exist between them. Secondly, the performance metrics used in this study, such as R-squared values, are likely to follow a normal distribution and exhibit homogeneous variances across groups. ANOVA is an appropriate statistical tool under these assumptions, as it relies on the normality of the data and the homogeneity of variances to produce valid results. Moreover, ANOVA results provide F-statistics and p-values, which clearly indicate whether the differences between group means are statistically significant. This clarity helps researchers quickly understand and interpret the results. Additionally, if ANOVA indicates significant differences, researchers can perform post-hoc tests to identify specifically which groups differ from each other.

### Experimental design

**Table 2:** Overview of analysis (MUT: mutation, CNV: Copy number variation)

	Analysis		
	1	2	3
Gene expression	$\vee$	$\vee$	$\vee$
Feature selection	$\vee$	$\vee$	$\vee$
Other omics(MUT, CNV)		$\vee$	
Drug group			$\vee$

The three analyses are presented in detail in Table 2. Firstly, the gene expression data from the GDSC dataset, which represents fundamental cellular state information for drug response, was employed to assess the precision of drug response prediction through the utilisation of various regression algorithms. In this analysis, the performance improvement of the regression model was evaluated by comparing the raw input features (RAW), which were used without preprocessing or feature selection, with data selected through feature selection methods (L1000, MI, SKB, VAR). The gene expression data from the GDSC dataset is organized in a matrix comprising 734 cancer cell lines and 8,046 genes (734 x 8046), which was used as the raw input.

The second analysis examined the influence of incorporating supplementary omics data, including mutation profiles and copy number variation (CNV) profiles, as input features alongside gene expression data on the regression model’s performance. This analysis compared the performance of using gene expression data alone (L1000), gene expression data with mutation profiles (L1000+MUT), gene expression data with CNV profiles (L1000+CNV), and gene expression data combined with both mutation and CNV profiles (L1000+MUT+CNV).

In the third analysis, the optimal input features identified in the first analysis (L1000) were employed to evaluate the prediction accuracy according to drug categories. The input data employed in each analysis was meticulously selected to align with the research objectives, thereby facilitating an assessment of the regression model’s performance and prediction accuracy.

## Results and discussion

### Effects of feature selection

**Table 3:** One-way ANOVA statistical analysis results (FS: Feature selection)

Algorithm	Best FS	ANOVA (F_statistic)	ANOVA (P-value)
CNN	RAW	13.38421269	1.25E-10
ADA	_	0.726724149	0.575
DTR	_	0.701805603	0.591
Elastic Net	RAW	35.58685083	7.99E-28
GBR	_	0.993721905	0.41
XGBR	_	0.932523202	0.444
GPR	RAW	68.72096921	2.00E-51
KNN	L1000	3.052226854	0.016
LASSO	L1000	5.377362062	0.00028
LGBM	_	0.764109533	0.549
MLP	L1000	101.6711437	1.16E-72
RFR	_	1.738450171	0.139
RIDGE	RAW	66.57878169	5.69E-50
SVR	L1000	6.049124373	8.24E-05

**Fig. 1:**
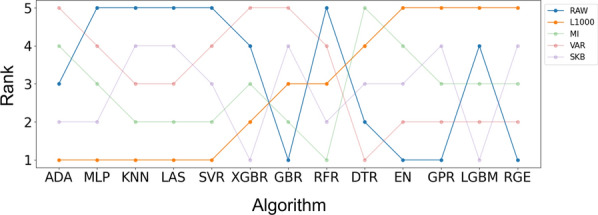
Ranking of feature selection algorithms using different regression methods

The selection of features is of paramount importance in enhancing the performance and efficiency of machine learning algorithms. The objective is to identify the optimal feature selection strategy through a comprehensive examination of a multitude of algorithms based on R-squared values. A one-way ANOVA test was employed to assess the outcomes of various algorithms, as illustrated in Table 3. Subsequently, the ranks were compared to ascertain the most effective feature selection technique (Fig. 1), namely RAW (no feature selection), L1000, MI, SKB, and VAR, as delineated in Sect. "Data sets".

**Fig. 2:**
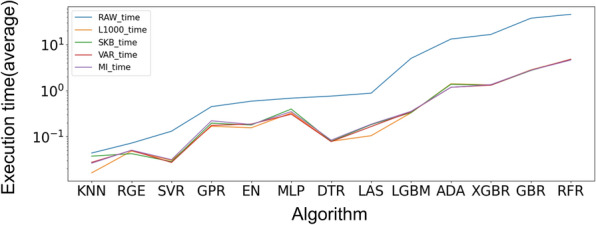
Execution time for feature selection algorithms with different regression methods

As demonstrated in Table 3, feature selection algorithms exhibited markedly disparate prediction accuracy across 8 out of 13 regression algorithms. In particular, the use of raw input features yielded the most favourable outcomes for the CNN, Elastic Net, GPR, and Ridge Regression methods. In contrast, the L1000 features yielded the most optimal outcomes for the KNN, LASSO, MLP, and SVR regression algorithms (Fig. 1). However, it was found that the use of raw input features resulted in a relatively lengthy execution time (Fig. 2). Therefore, L1000 was selected as the final feature selection strategy for the subsequent experiments, given its superior performance and reduced time consumption. The selected technique not only ensures the effective utilisation of computational resources but also maintains the accuracy of the predicted outcomes, thereby representing the optimal option for the algorithms under consideration.

### Effects of multiomics information

**Fig. 3:**
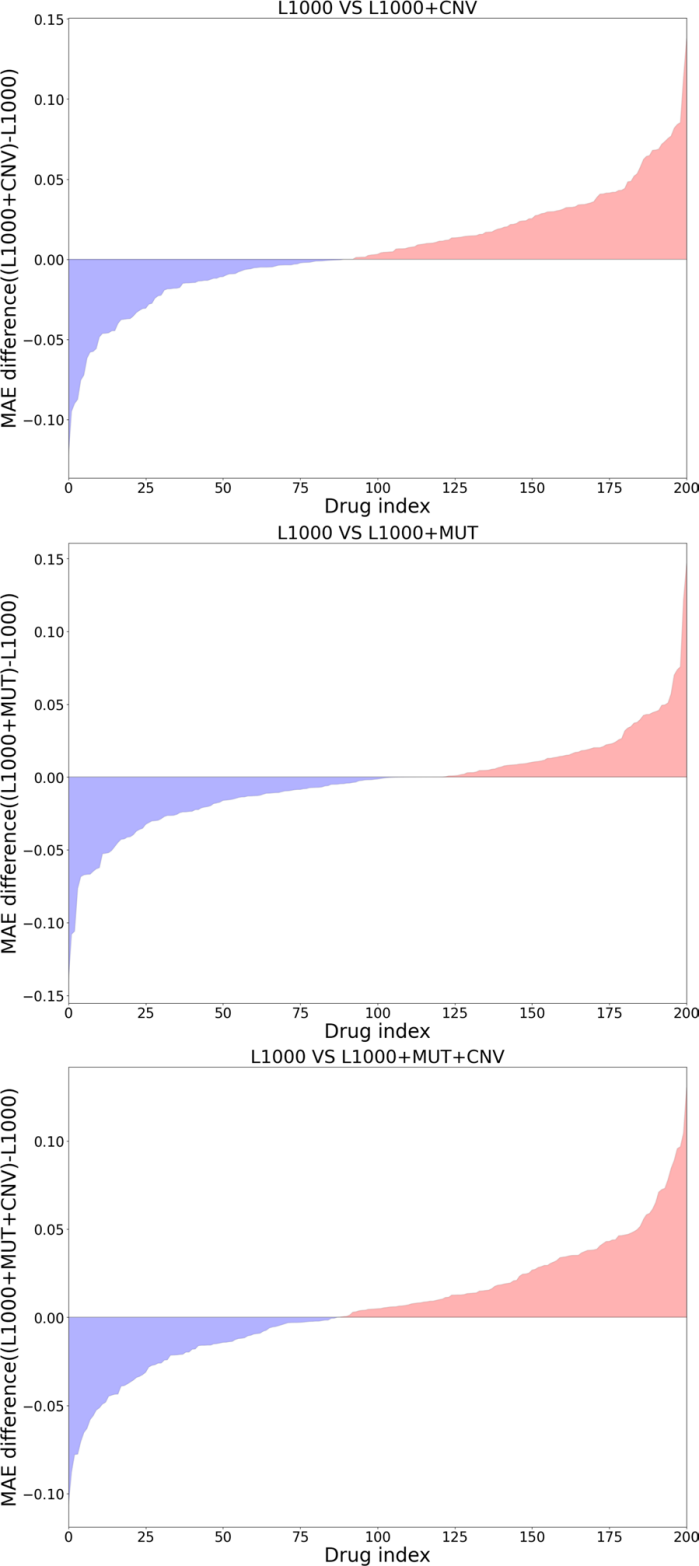
Relative predictive performance when training with gene expression features alone (L1000) vs. using additional mutation (MUT) and copy number variation (CNV) features

The objective was to ascertain whether integrating mutation (MUT) and copy number variation (CNV) data with the L1000 dataset yields discernible differences in predictive modelling performance. To investigate this, the results from each combination (L1000+MUT, L1000+CNV, and L1000+MUT+CNV) were visualised and independent t-tests were used to determine the statistical significance of any detected changes. The objective of this experiment was to identify potential performance gaps before and after the integration of other omics data, and to determine whether these differences were statistically significant (Fig. 3).

Our findings revealed an intriguing result: there were no statistically significant differences between the L1000 (only gene expression data) and the L1000+MUT, L1000+CNV, and L1000+MUT+CNV datasets. In light of the aforementioned findings, it can be concluded that the incorporation of additional features derived from mutation or copy number variation did not result in a notable enhancement in prediction accuracy. Nevertheless, a subsequent study could be conducted to ascertain whether the incorporation of additional feature selection or extraction for multi-omics data could enhance the performance further.

**Fig. 4:**
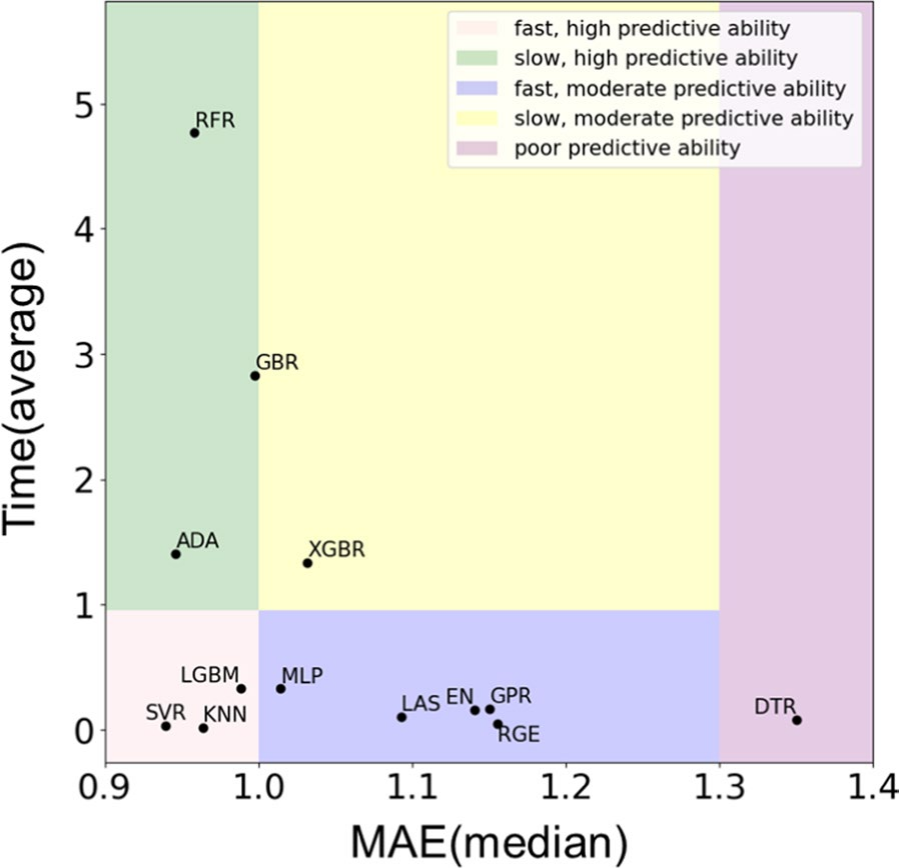
Execution time and predictive performance for regression algorithms in L1000+MUT dataset

The performance variances among the 13 algorithms used to process the L1000 dataset were examined using scatter plots and boxplots. The horizontal axis of the scatter plot represents the mean absolute error (MAE) score, while the vertical axis depicts the corresponding calculation time (Fig. 4). An evaluation of the calculation time was conducted using a time scale of 1 s, which has been widely accepted as a benchmark in various interactive and real-time applications. This provided a practical threshold for classifying the speed of the algorithms as either ‘fast’ or ‘slow’. The algorithms were thus classified into five distinct regions: (fast, high predictive skills), (quick, low predictive abilities), (slow, high predictive abilities), (slow, low predictive abilities), and (bad predictive abilities). The combination of these two measures indicates that the SVR, KNN, and LGBM algorithms demonstrate superior performance in the (L1000+MUT) dataset.

**Fig. 5:**
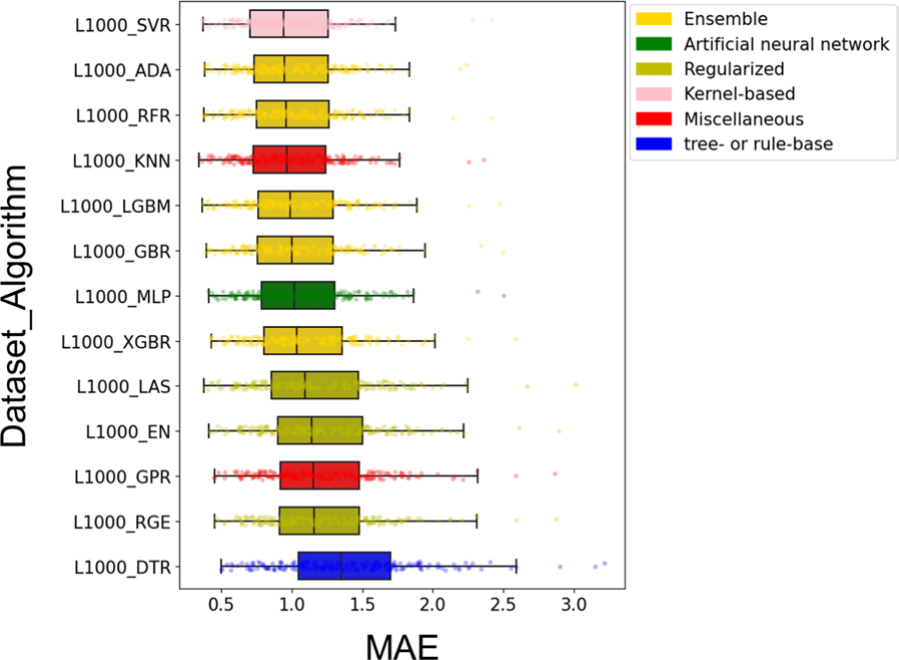
A boxplot that simultaneously expresses the performance of each algorithm in L1000+MUT dataset

As illustrated in Fig. 5, ensemble algorithms demonstrate superior performance compared to other groups. The boxplots effectively illustrate the distribution of performance measurements while emphasising the remarkable predictive accuracy and robustness of ensemble algorithms. The results presented herein demonstrate the effectiveness of ensemble approaches in combining the strengths of various algorithms to provide more accurate predictions. The visual proof provided by the boxplots demonstrates that ensemble algorithms are capable of consistently outperforming their competitors in a range of predictive tasks.

Furthermore, our findings indicate that the Support Vector Regression (SVR) algorithm exhibited superior predictive performance compared to the other algorithms. It is noteworthy that SVR exhibited exemplary predictive performance while necessitating a markedly reduced training period. This combination of high performance and efficiency establishes SVR as the leading algorithm among those under consideration.

Furthermore, to gain additional insight into the individual impact of MUT and CNV data on drug response prediction, an additional experiment was conducted using only MUT and CNV data as input features. The results of this experiment are presented in supplementary file (Additional file 1 Figure S1), and this analysis serves to isolate the specific contributions of these omics data to predictive modelling. However, it was observed that the performance was not satisfactory when using only MUT and CNV data.

### Performance comparison between drug groups

The objective of this study was to investigate the performance differences of the SVR algorithm when applied to the L1000 dataset, with a particular focus on different drug groups. The mean absolute error (MAE) scores were evaluated, and the findings were displayed through a series of boxplots after the dataset was segmented according to the drug’s functional categories.

**Fig. 6:**
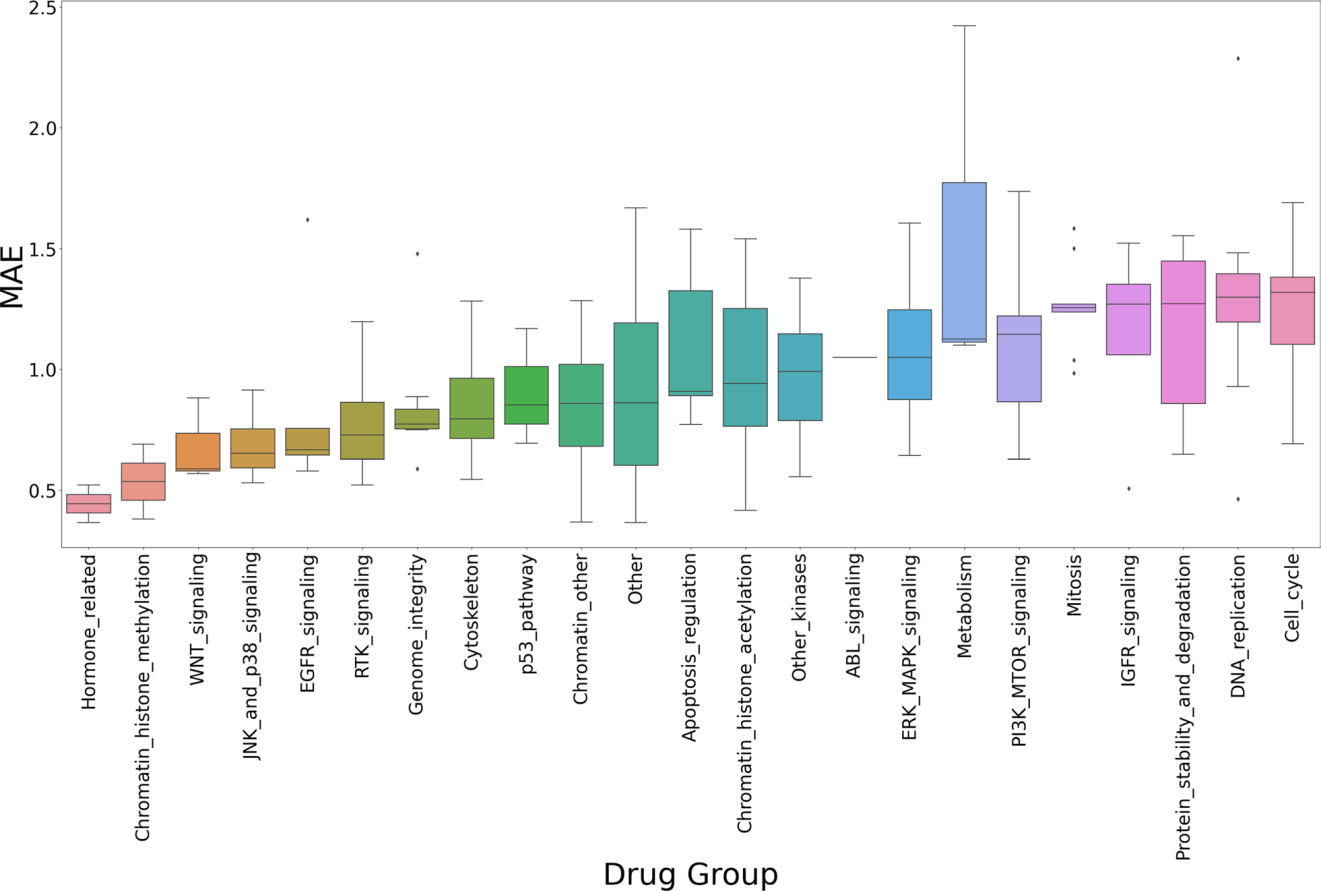
Predictive performance of SVR with L1000 features for each drug group

As illustrated in Fig. 6, the notable performance disparity observed among the drug group associated with the HORMONE-RELATED PATHWAY is particularly noteworthy. Conversely, our analysis indicates that SVR-based forecasts within this particular drug group exhibited elevated MAE scores, suggesting diminished predictive precision. The discrepancy in performance across drug groups underscores the intricate relationship between biological pathways and predictive modelling outcomes.

## Conclusion

In this study, drug response prediction was conducted using 13 representative regression algorithms and the large-scale GDSC dataset. Three analyses were designed to demonstrate the impact of feature selection methods, multiomics information, and drug categories on drug response prediction. In the initial experiment, a comparison was made between four feature selection algorithms. The 627 gene features selected with the L1000 dataset were found to demonstrate the optimal performance in terms of accuracy and execution time. The incorporation of mutation and copy number variation data did not yield enhanced prediction accuracy relative to the utilisation of gene expression data alone. Among the drug groups, the responses of drugs associated with the hormone pathway were predicted with a relatively high degree of accuracy. The Support Vector Regression algorithm exhibited superior performance in the majority of experiments, as evaluated by two criteria: accuracy and time.

These findings not only demonstrate the efficacy of specific regression algorithms but also underscore the potential for further refinement and enhancement through the integration of additional data sources and methodologies. The utilisation of the GDSC dataset with readily accessible regression models enables the formulation of guidelines pertaining to data preprocessing, integration, and algorithm selection, thereby providing valuable insights for researchers engaged in the fields of biology and bioinformatics. The experimental results suggest that the accuracy of drug response prediction may be enhanced by the utilisation of additional information sources beyond gene expression data. This may entail the incorporation of high-throughput screening data from the LINCS L1000 dataset to identify salient features. Notwithstanding the limited impact of integrating other omics data on drug response prediction, we propose the proposition that a more sophisticated integration of multiomics data with feature extraction and hyperparameter tuning may serve to enhance the accuracy of the predictions, as demonstrated in the subsequent study.

Building on these insights, future research can further extend the scope of this study by incorporating additional datasets and advanced methodologies. While the present study offers substantial insights into drug response prediction using the GDSC dataset, there are several promising avenues for further investigation and refinement, such as the Cancer Cell Line Encyclopedia (CCLE). The integration of CCLE data with GDSC has the potential to yield several important advantages. Firstly, the combination of both datasets would allow for the validation of findings across disparate data sources, thereby enhancing the reliability and reproducibility of the predictive models. Secondly, the incorporation of CCLE data would enhance the diversity of cell lines and drugs analysed, thereby facilitating the development of more generalised and robust models. Thirdly, a comparative analysis of the CCLE and GDSC datasets could reveal consistent patterns and complementary insights, thereby facilitating more accurate predictions. Lastly, the discrepancies observed between the datasets could provide new insights into biological processes, thereby deepening our understanding of cancer treatment and contributing to the development of more effective therapeutic strategies.

## Supplementary Information

Supplementary material 1.

## Data Availability

The GDSC data used in the study are downloaded from https://github.com/Jinyu2019/Suppl-data-BBpaper.

## Abbreviations

ANOVA: Analysis of variance
CCLE: Cancer cell line encyclopedia
CNV: Copy number variation
FS: Feature selection
GDSC: Genomics of Drug Sensitivity in Cancer
KNN: K-nearest neighbour
LINCS: Library of integrated network-based cellular signatures
MAE: Mean absolute error
MI: Mutual information
MSE: Mean squared error
MUT: Mutation
RMSE: Root mean squared error
SKB: Select K best features
SVR: Support vector regression
VAR: Variance threshold
