# Supplementary material for: Comparative analysis of regression algorithms for drug response prediction using GDSC dataset
Source: BMC Res Notes. 2025 Jan 13;18(Suppl 1):10. doi: 10.1186/s13104-024-07026-w (PMC11726955; doi:10.1186/s13104-024-07026-w)
Supplement: Supplementary file 1 — Supplementary material 1. [file 13104_2024_7026_MOESM1_ESM.pdf]

Supplementary Information for "Comparative Analysis of Regression Algorithms for Drug Response Prediction Using GDSC Dataset"

Soojung Ha, Juho Park, Kyuri Jo

1 Supplementary Figure

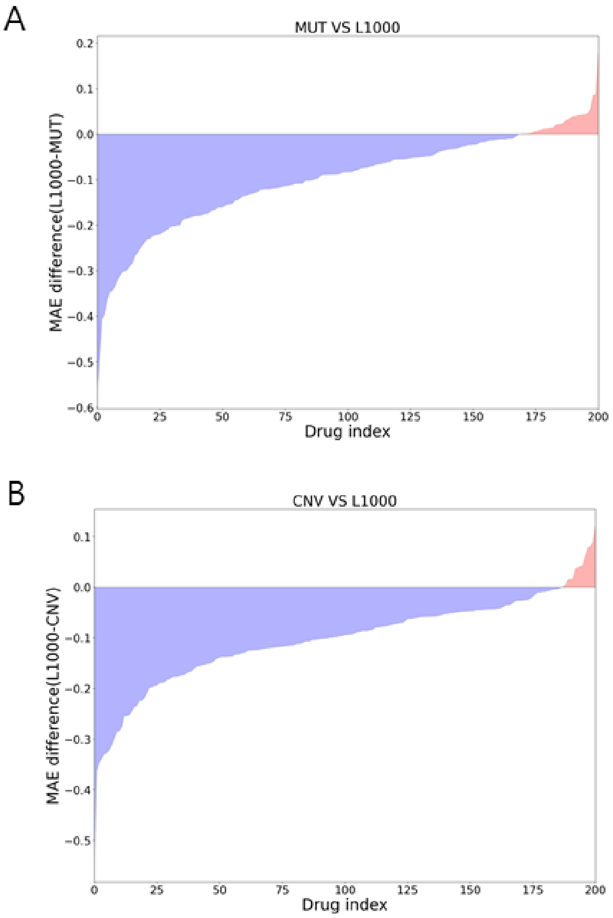

Figure S1: A comparative analysis of the predictive performance of gene expression features (L1000) versus mutation (MUT) and copy number variation (CNV) features alone. (A) The graph illustrates the discrepancy in mean absolute error (MAE) between the utilization of MUT features and L1000 features, calculated by subtracting the MUT MAE from the L1000 MAE. (B) The graph depicts the MAE difference between the application of CNV features and L1000 features, calculated in a similar manner by subtracting the CNV MAE from the L1000 MAE.

2 Supplementary Table

Table S1: The table presents a comprehensive overview of the diverse array of machine learning models employed for regression, with a particular emphasis on the specific parameters associated with each model. It includes the following: Model, The specific machine learning algorithm utilized in the study. Default Parameters, The parameters that are set by default when the model is initialized without any specific configuration. Additional Specified Parameters, These are the parameters that were explicitly defined or modified by the researchers in order to better tune the model for the given data set or experiment. The final parameters utilized in the study, The parameters that were ultimately selected and utilized following the application of techniques such as GridSearchCV or based on the specific experimental conditions.

Model	Default Parameters	Additional Specified Parameters	Final Used Parameters
KNeighborsRegressor	n_neighbors=5, leaf_size=30, weights=uniform, algorithm=auto, p=2, metric=minkowski	n_neighbors=[4, 5, 6], leaf_size=[25, 30, 35]	n_neighbors=6, leaf_size=25
RandomForestRegressor	n_estimators=100, criterion=squared_error, max_depth=None, min_samples_split=2, bootstrap=True	n_estimators=[70, 100, 130], min_samples_split=[1, 2, 3]	n_estimators=130, min_samples_split=3
SVR	kernel=rbf, C=1.0, epsilon=0.1, gamma=scale	kernel=[linear, poly, rbf]	kernel=rbf
DecisionTreeRegressor	criterion=squared_error, splitter=best, max_depth=None, min_samples_split=2	criterion=[squared_error, friedman_mse, absolute_error, poisson]	criterion=friedman_mse
AdaBoostRegressor	n_estimators=50, learning_rate=1.0, loss=linear	n_estimators=[40, 50, 60], learning_rate=[0.05, 0.1, 0.15]	n_estimators=60, learning_rate=0.15

GradientBoostingRegressor	learning_rate=0.1, n_estimators=100, subsample=1.0, max_depth=3, min_samples_split=2	learning_rate=[0.05, 0.1, 0.15]	learning_rate=0.05
LGBMRegressor	num_leaves=31, max_depth=-1, learning_rate=0.1, n_estimators=100, subsample=1.0	num_leaves=[24, 31, 38], max_depth=[-1, 20, 30]	num_leaves=24, max_depth=-1
XGBRegressor	objective=reg	max_depth=[5, 6, 7]	max_depth=5
MLPRegressor	hidden_layer_sizes=(100,), activation=relu, solver=adam, alpha=0.0001, max_iter=200	hidden_layer_sizes=[5, 10, 15], max_iter=[100, 150, 200]	hidden_layer_sizes=15, max_iter=200, random_state=0
GaussianProcessRegressor	kernel=None, alpha=1e-10, optimizer=fmin_l_bfgs_b, n_restarts_optimizer=0, normalize_y=False	alpha=[1e-14, 1e-10, 1e-6]	kernel=DotProduct() + WhiteKernel(), alpha=1e-6, random_state=0
Ridge	alpha=1.0, fit_intercept=True, normalize=False, solver=auto	alpha=[0.01, 0.02, 0.03]	alpha=0.03
Lasso	alpha=1.0, fit_intercept=True, normalize=False, max_iter=1000, tol=0.0001	alpha=[0.01, 0.02, 0.03]	alpha=0.03
ElasticNet	alpha=1.0, l1_ratio=0.5, fit_intercept=True, normalize=False, max_iter=1000	alpha=[0.01, 0.02, 0.03]	alpha=0.03, random_state=0
